# History based forward and feedback mechanism in cooperative spectrum sensing including malicious users in cognitive radio network

**DOI:** 10.1371/journal.pone.0183387

**Published:** 2017-08-18

**Authors:** Noor Gul, Ijaz Mansoor Qureshi, Adnan Omar, Atif Elahi, Sajjad Khan

**Affiliations:** 1 Department of Electrical Engineering, International Islamic University, Islamabad, Pakistan; 2 Department of Electrical Engineering, Air University, Islamabad, Pakistan; 3 Institute of signals, systems and soft computing (ISSS), Islamabad, Pakistan; University of Rijeka, CROATIA

## Abstract

In cognitive radio communication, spectrum sensing plays a vital role in sensing the existence of the primary user (PU). The sensing performance is badly affected by fading and shadowing in case of single secondary user(SU). To overcome this issue, cooperative spectrum sensing (CSS) is proposed. Although the reliability of the system is improved with cooperation but existence of malicious user (MU) in the CSS deteriorates the performance. In this work, we consider the Kullback-Leibler (KL) divergence method for minimizing spectrum sensing data falsification (SSDF) attack. In the proposed CSS scheme, each SU reports the fusion center(FC) about the availability of PU and also keeps the same evidence in its local database. Based on the KL divergence value, if the FC acknowledges the user as normal, then the user will send unified energy information to the FC based on its current and previous sensed results. This method keeps the probability of detection high and energy optimum, thus providing an improvement in performance of the system. Simulation results show that the proposed KL divergence method has performed better than the existing equal gain combination (EGC), maximum gain combination (MGC) and simple KL divergence schemes in the presence of MUs.

## Introduction

The demand for radio spectrum is on the rise and is considered asa serious issue. Cognitive Radio Network recently emerged as the prime method for the efficient utilization of flexible radio spectrum[1]. The key responsibility of the Cognitive Radio Networks is sensing spectrum of the primary users(PUs), dynamic spectrum access, spectrum management and efficient spectrum utilization. In spectrum sensing, secondary users (SUs) monitor the activities of the PUs to find out spectrum holes for the transmission of SUs without any interference to the PUs[2–4].

To obtain information about the existence of PU, one of the detection schemes such as generalized likelihood ratio test detector(GLRT), matched filter detector(MFD), feature detector or energy detectors may be used. The GLRT scheme takes some of the system parameters, such as channel gains, noise variance and PU signal variance as unknown parameters[5–8]. MFD is an optimal detection method if information about the PU signal is already obvious to the SU[9]. Feature detector uses cyclo-stationary features of the signal and has better performance in the case of weak signals at low signal to noise ratio (SNR)[10,11]. Energy detector is the most efficient scheme in case the cognitive radio has limited or deficient information about the PU signals [9,12,13].

Sensing radio spectrum of the PU is highly influenced by fading, shadowing and receiver uncertainty problems between PU and SUs[13–15]. Therefore, sensing results may not be considered reliable in case of single SU. This problem is overcome by taking advantages of cooperative spectrum sensing (CSS), where each SU collects information about the presence of PU and share its local sensing information with fusion center (FC) to generate a combined solution about the existence of PU with improved detection results[16–20]. The cooperation process between SUs can be centralized or distributed[21]. Two hard CSS combinations OR and AND were studied in[22,23]. Counting rule and voting rules are discussed in[13,23–27]. Instead of hard combinations schemes there are some soft combination methods, such as maximum gain combination (MGC) and equal gain combination (EGC)[28]. Contrary to the hard combination in which SU sends single bit information to the FC, in soft schemes, each SU sends entire sensing information to the FC and earn more precise spectrum sensing performance than hard decision schemes[29–31].

Problem occurring in CSS is the existence of malicious user (MU).MU sends false data which misdirect other SUs about the deficiency of the spectrum and may stop them from using the free channel or giving wrong oversight about the spectrum sufficiency, while it is already in use of the PU. The existence of such misbehaving users degrade the performance of CSS[32]. To minimize the effects of spectrum sensing data falsification (SSDF) users, a robust outlier detection scheme is proposed in[33,34]. A CSS based on the sensing performance of each SU is discussed in[35–38].The SSDF attack of always Yes, always No and random MUs are studied in[39–41]. Statistical methods employed by MUs for attack are analyzed in[42,43].An abnormality based detection approach is applied in[44,45] to oppose the unknown tactic of the attacker.

In the processes of soft combination schemes, MGC is the optimal choice. However, this scheme requires information between the SU and the PU, which is difficult to get in practice. The MGC is also sensitive to the attack of MUs providing false sensing data to the FC[17]. The CSS protection scheme, discussed in[33,34]is used for the detection of always Yes and always No MUs, where outlier detection technique is used. However performance of the proposed method in[33,34] is highly reduced in case of large number of MUs.Kullback-Leibler (KL) divergence based CSS is proposed in[12] to protect the CSS against large number of MUs with less computational complexity unlike the method in[33,34].

In this paper, a change is proposed in the sensing procedure of the CSS scheme using KL divergence as discussed in[12]. In[12] SUs send energy information to the FC without storing and receiving any reputation feedback information from the FC regarding the KL divergence score. In the proposed work SUs sense the PU spectrum, store and forward its energy information to the FC. Based on the received energy reports from each SU, FC measures individual KL divergence value for each SU and sends this information back to SUs and also takes a global decision about the PU spectrum. An SU with achieved KL divergence limits from the FC, will compute the mean values of the energy statistics reported under both free and occupied status of the PU. In the following sensing intervals the SUs observe the channel behavior and forward this average statistics information to the FC. The decision to forward either average free or occupied energy statistics is based on the current sensing observation of the channel.

The proposed method is tested against the existence of always Yes, always No, opposite and random opposite MUs. It is exhibited that this change in the sensing and reporting procedure results in more accurate and sophisticated detection of the PU as compared to the traditional KL divergence, EGC and MGC schemes as in [12,17] with optimum energy consumption by each SU.

The rest of the paper is organized as follows. In Section 2, the system model is explained. Section 3 addresses how FC utilizes the individual energy reports of all SUs to generate global decision of the PU detection and sends back the individual KL divergence values to each SU. Experimental results are presented in Section 4. Section 5 concludes the paper.

## Data model

**Fig 1:**
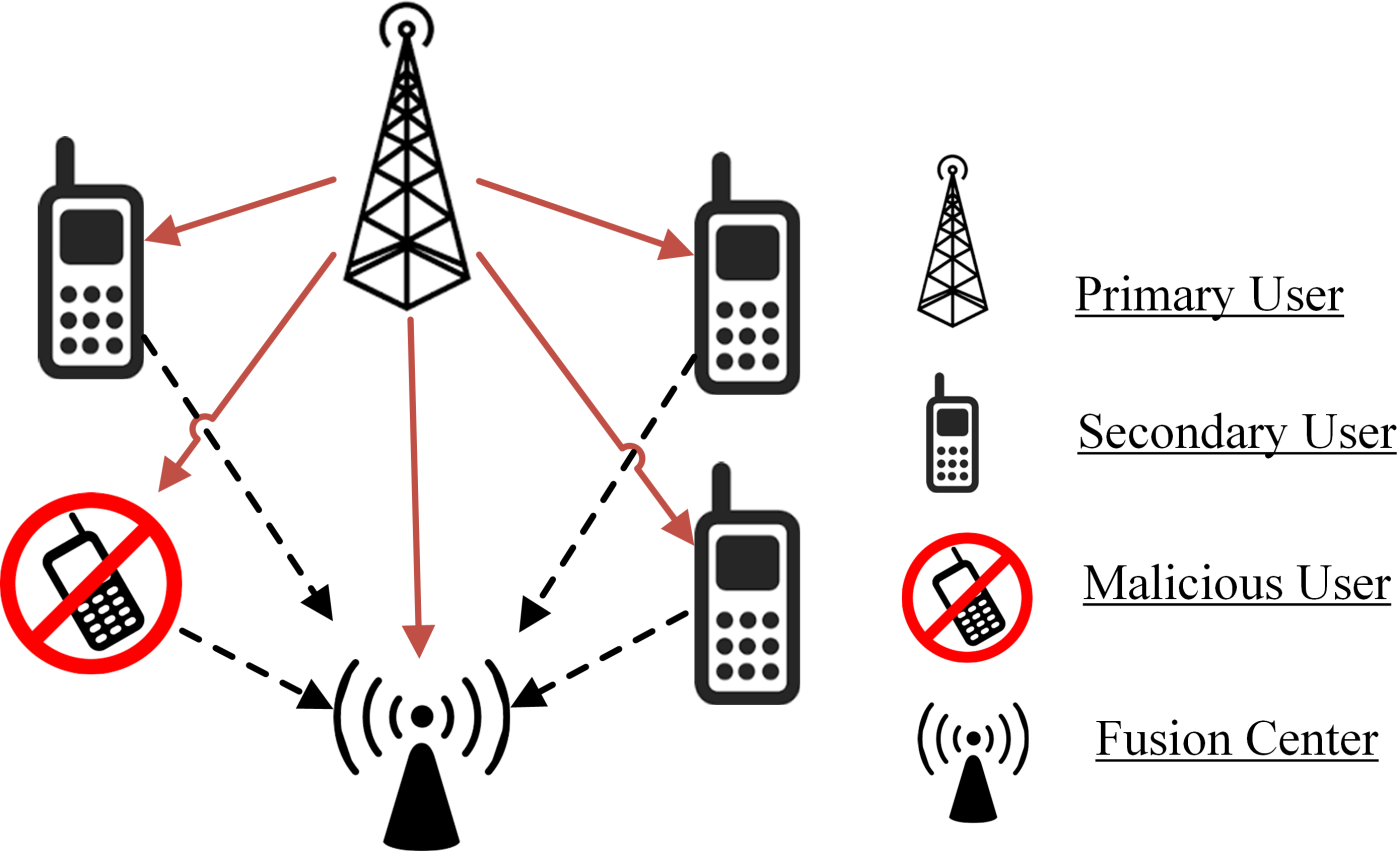
Conventional CSS mechanism.

All SUs in the centralized CSS as in Fig 1 report FC about the existence of PUs with local spectrum sensing information. FC combines the received sensing notifications from all SUs with his own sensing results and generates a global decision about the free and the occupied status of the PU spectrum.

Based on the spectrum sensing information by each SU in a particular band decision between *H*_1_ and *H*_0_ isas follows:

$$y_{j}(l)=\left\{\begin{array}{l} H_{0},\;n_{j}(l)\\ H_{1},\;h_{j}s(l)+n_{j}(l) \end{array}\right\}$$ (1)

Where *H*_0_ and *H*_1_ are the hypothesis about the absence and presence of the PU. *y*_*j*_(*l*) is the received signal from the *j*^*th*^ SU, *n*_*j*_ (*l*) is the additive white gaussian noise at the *l*^*th*^ time slot for the *j*^*th*^ SU, *h*_*j*_ is the channel gain value between the *j*^*th*^ SU and PU and *s*(*l*) is the signal transmitted from the PU. According to the hypothesis *H*_1_ and *H*_0_ the received signal energy of the channel by the *j*^*th*^ SU user at the *i*^*th*^ sensing interval is:

$$E_{j}(i)=\left\{\begin{array}{l} \sum_{l=l_{i}}^{l_{i}+M-1}{\left|n_{j}(l)\right|}^{2},\;H_{0}\\ \sum_{l=l_{i}}^{l_{i}+M-1}{\left|h_{j}s(l)+n_{j}(l)\right|}^{2},\;H_{1} \end{array}\right\}$$ (2)

where *M* is representation of the number of samples in the *i*^*th*^ sensing interval. The number of samples *M* is to be considered large enough such that the energy reported by each SU resembles a gaussian random variable under both *H*_0_ and *H*_1_ hypothesis[46].

$$E_{j}∼\left\{\begin{array}{l} N\left(\mu_{0}=M,\sigma_{0}^{2}=2M\right),\;H_{0}\\ N\left(\mu_{1}=M(\eta_{j}+1),\sigma_{1}^{2}=2M(\eta_{j}+1)\right),\;H_{1} \end{array}\right\}$$ (3)

Here *η*_*j*_ is the SNR value between the *j*^*th*^ SU and the PU. $\left(\mu_{0},\sigma_{0}^{2}\right)$, $\left(\mu_{1},\sigma_{1}^{2}\right)$ are the mean and variance values of the energy under *H*_0_ and *H*_1_ hypothesis.

As the KL divergence value between the two probability distribution functions(PDFs) *a*(*x*) and *b*(*x*) both normally distributed is calculated as follows[47].

$$K\left(a\|b\right)=\int a(x)\log\left[\frac{a(x)}{b(x)}\right]dx$$ (4)

Similarly, the KL divergence for functions *a*(*x*) with mean and variance $\left(\mu_{a},\sigma_{a}^{2}\right)$ and function *b*(*x*) with mean and variance values $\left(\mu_{b},\sigma_{b}^{2}\right)$ is further calculated as:

$$\begin{array}{l} K\left(a\|b\right)=K\left(\mu_{a},\mu_{b},\sigma_{a}^{2},\sigma_{b}^{2}\right)\\ \;=\frac{1}{2}\left[\log\left(\frac{\sigma_{b}^{2}}{\sigma_{a}^{2}}\right)-1+\frac{\sigma_{a}^{2}}{\sigma_{b}^{2}}+\frac{{\left(\mu_{a}-\mu_{b}\right)}^{2}}{\sigma_{b}^{2}}\right] \end{array}$$ (5)

**Fig 2:**
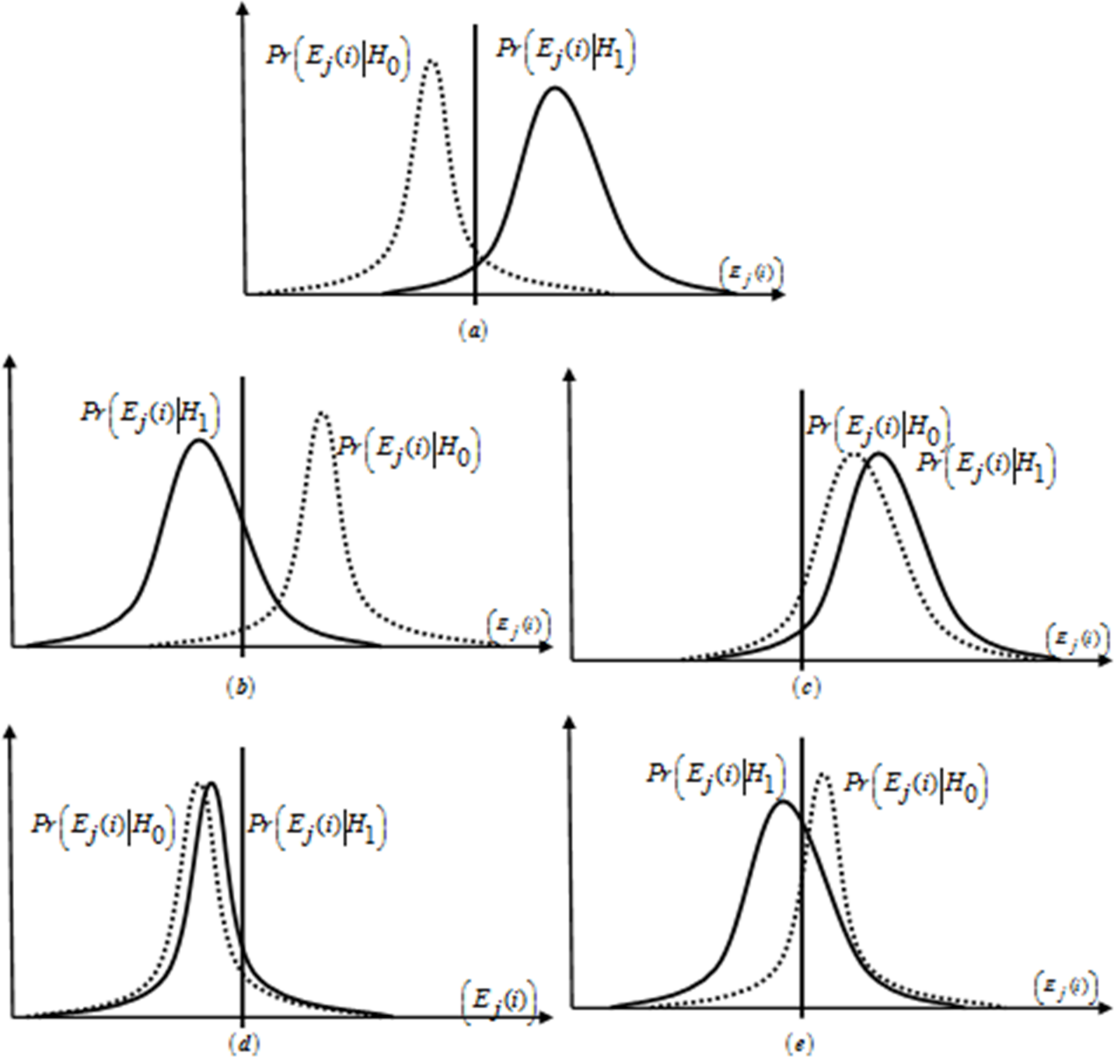
PDF of the energy distribution reported from the CR users under the absence or presence hypothesis of the PU signal: (a) nomal user, (b) opposite MU, (c) always Yes MU, (d) always No MU, (e) random opposite MU.

The result in Eqn.(5) clearly shows that for functions *a*(*x*) and *b*(*x*) with similar PDF occurrence, has “0”KL divergence value.As always Yes and always No MUs are giving identical energy distribution with similar mean and variance values under both *H*_1_ and *H*_0_ as in Fig 2, thereforethe KL divergence has a value 0 foralways Yes and always No MU. The opposite MU and random opposite MUs is generating dissimilar KL divergence results in comparison with normal SUs as shown in the energy distribution of Fig 2.

The PDF of the energy distributions received from the normal SU, opposite MU, always Yes MU, always No MU and random opposite MU is shown in Fig 2 for comparison. It is to be noted that the energy distribution of these MUsis totally different from the normal SU. These differences in the energy distribution of the SUs is used for the detectionof MUs. A normal SU in Fig 2A is shown with positive energy distribution under *H*_1_ hypothesis and negative energy distribution under *H*_0_ hypothesis. The opposite MU have opposite energy distribution to the normal users as in Fig 2B under both *H*_0_ and *H*_1_. Always Yes MUs with positive energy distribution under both *H*_0_ and *H*_1_ hypothesis and always No MUswith negative energy distribution under both *H*_0_ and *H*_1_ hypothesis are shown in Fig 2C and 2D.Random opposite MU has statistically opposite nature to the normal SUs with probability *p* and results in distributions as in Fig 2E in both hypothesis.

## Proposed algorithm for the detection of MUs based on Kullback-Leibler divergence

**Fig 3:**
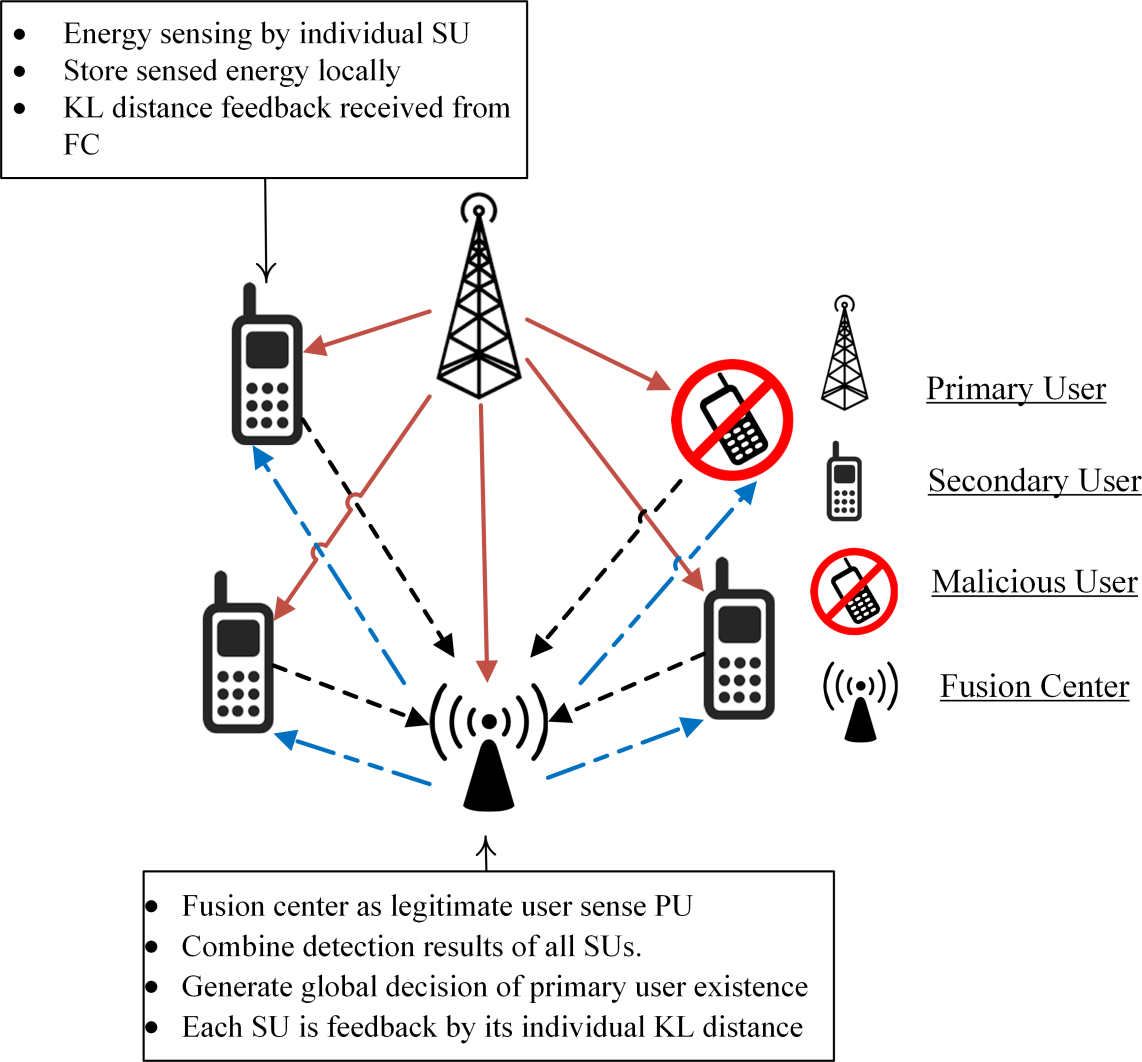
Proposed CSS mechanism.

In the proposed work, the total number of MUs considered is less than the total number of cooperating SUs. All SUs report FC about the existence of PUs with local spectrum sensing information and also stores this data locally.FC combines the individual reports and generate a global decision of the PU spectrum. FC also creates a feedback report for each SU about its individual detection performance as in Fig 3 by measuring the KL divergence score for each SU.Before SU reports any sensing information, it compares the detection results feedback received from the FC with a target value. Based on the feedback from the FC, if the detection results are achieved on behalf of a user,then this particular user will further participate in the sensing process by combining current sensing results with its local history to report a more solid PU status to FC. SUs not declaredas normal will forward their current sensing energy of the PU channel to the FC, while theconfirmednormal user will sense the channel and forward meanenergy of the reports already made under *H*_1_ and *H*_0_ hypothesis.

### Pseudo code of the proposed method is as below

- For *i* = 1 to sensing limit
  ○ For *j* = 1 to number of SU
    ○ IF (∑_*i*_*K*_*j*_(*i*−1)<*T*_1_) OR (*i* = 1)
          *Z*_*j*_(*i*) *= E*_*j*_(*i*). Where *E*_*j*_(*i*) is current sensing energy
    ○ Else
          *Z*_*j*_(*i*) *= M*_1*j*_(*i*) *or M*_0*j*_(*i*), where *M*_1*j*_(*i*) and *M*_0*j*_(*i*) is the average of the reported energy for the *j*^*th*^ SU under *H*_1_ and *H*_0_ Hypothesis.
    ○ End IF
  ○ End of loop
  ○ For *j* = 1 to number of SU
  ○ Estimate new values of mean and variances under *H*_1_ as $\left(\mu_{j1\_new},\sigma_{j1\_new}^{2}\right)$ and under *H*_0_ as $\left(\mu_{j0\_new},\sigma_{j0\_new}^{2}\right)$ based on *Z*_*j*_(*i*)
  ○ Difference of the KL distances for the *j*^*th*^ SU under *H*_1_ and *H*_0_ is measured as Δ*K*_*L*,*j*_(*i*).
  ○ Update the KL distance score *K*_*j*_(*i*) for the *j*^*th*^ SU as $K_{j}(i)=\sum_{i}^{N}K_{j}(i-1)+\Delta K_{L,j}(i)$ and send feedback report of *K*_*j*_(*i*) to the *j*^*th*^ SU.
  ○ End of loop
  ○ The combine KL divergence is determined as Δ*K*_*T*_(*i*) = ∑_*j*_*W*_*j*_ × Δ*K*_*L*,*j*_(*i*). Where *W*_*j*_ is the weighting factor assigned to the *j*^*th*^ SUs decision.
  ○ IF Δ*K*_*T*_(*i*) > 0
    G_B_(i) = 1
  ○ Else
        G_B_(i) = 0
  ○ End IF
  ○ IF *G*_*B*_*(i) = 1*
        Update mean *μ*_*j*1_ and variance $\sigma_{j1}^{2}$ for next iteration.
  ○ Else
        Update mean *μ*_*j*0_ and variance $\sigma_{j0}^{2}$ for next iteration.
  ○ End IF
- End sensing limit

### Local decision and history maintenance by SU

In this step pre-sensing check is done by each SU, before forwarding, local sensing information to the FC based on its KL distance feedback information received from the FC.

$$Z_{j}(i)=\left\{\begin{array}{l} E_{j}(i),\;IF\left(i=1\right)OR\left(\sum_{i}K_{j}(i-1)<T_{1}\right)\\ M_{1j}(i)\;or\;M_{0j}(i),\;Otherwise \end{array}\right\}$$ (6)

Where ∑_*i*_*K*_*j*_(*i* − 1) is the KL distance value received by the *j*^*th*^ SU and *M*_1*j*_(*i*), *M*_0*j*_(*i*) are the mean sample values of all sensing energies reported by the *j*^*th*^ SUs under *H*_1_ and *H*_0_ hypothesis based on the history results.

If it is the first time, sensing is done by the *j*^*th*^ SU or if the KL divergence satisfaction score is not achieved by a particular SU then, according to Eqn.(6) the sense energy *Z*_*j*_(*i*) = *E*_*j*_(*i*) is reported by the SU to the FC and stores this energy locally for future implication.

Similarly, if detection results for the *j*^*th*^ SU is met by achieving the KL divergence satisfaction score, then the user is declared as normal. The normal user will search local history and calculate the mean of all high reporting energies as *M*_1*j*_(*i*) and of low energies as *M*_0*j*_(*i*) and will no more send energy *E*_*j*_(*i*) to the FC as:

$$Z_{j}(i)=\left\{M_{1j}(i)\;or\;M_{0j}(i),\;IF\left(\sum_{i}K_{j}(i-1)\geq T_{1}\right)\right\}$$ (7)

The normal SUs further forward these mean energy samples to the FC during the current and in the following sensing intervals according to the observation of the channel to forward decision *M*_1*j*_ or *M*_0*j*_ to the FC.

### KL divergence at the FC

Based on the energies reported by the *j*^*th*^ SU and the previous mean and variance values, new values of the mean and variances in the *i*^*th*^ sensing interval is calculated for all SUs at the FC as follows:

$$\begin{array}{l} \mu_{j1\_new}(i)=z_{1}\mu_{j1}+z_{2}Z_{j}(i)\\ \sigma_{j1\_new}^{2}(i)=z_{1}\sigma_{j1}^{2}+z_{1}{\left[Z_{j}(i)-\mu_{j1}\right]}^{2}\\ \mu_{j0\_new}(i)=z_{1}\mu_{j0}+z_{2}Z_{j}(i)\\ \sigma_{j0\_new}^{2}(i)=z_{1}\sigma_{j0}^{2}+z_{1}{\left[Z_{j}(i)-\mu_{j0}\right]}^{2} \end{array}$$ (8)

*z*_1_ and *z*_2_ are constants with $z_{1}=\frac{k-1}{k}$ and $z_{2}=\frac{1}{k}$. Here *k* is the effecting level of the received energy to corresponding mean and variance of SUs PDF.

The KL divergence value for the *j*^*th*^ SU is determined as:

$$K_{j1}(i)=KL\left[\mu_{j1\_new}(i),\mu_{j1},\sigma_{j1\_new}^{2}(i),\sigma_{j1}^{2}\right]$$ (9a)

$$K_{j0}(i)=KL\left[\mu_{j0\_new}(i),\mu_{j0},\sigma_{j0\_new}^{2}(i),\sigma_{j0}^{2}\right]$$ (9b)

Where *K*_*j*1_(*i*) is the KL divergence under the presence hypothesis for the *j*^*th*^ SU and *K*_*j*0_(*i*) is the KL divergence for the *j*^*th*^ SU under absence hypothesis. Difference in the probability distribution function Δ*K*_*L*,*j*_(*i*) for the *j*^*th*^ SU under *H*_1_ and *H*_0_ hypothesis is calculated as:

$$\Delta K_{L,j}(i)=(K_{j1}(i)-K_{j0}(i))$$ (10)

The total KL divergence value *K*_*j*_(*i*) of the *j*^*th*^ user is further updated as below:

$$K_{j}(i)=\sum_{i}K_{j}(i-1)+\Delta K_{L,j}(i)$$ (11)

This updated value of *K*_*j*_(*i*) is sent by the FC to the *j*^*th*^ SU in order to utilize this information prior to any further reports.

### Global decision at the FC

Based on the KL divergence values of all SUs, the global decision *G*_*B*_(*i*) is made at the FC as follows:

$$G_{B}(i)=\left\{\begin{array}{l} H_{1},\;IF(\Delta K_{T}(i)=\sum_{j}W_{j}\times \Delta K_{L,j}(i))\leq 0\\ H_{0},\;Otherwise \end{array}\right\}\;where\;W_{j}=\frac{1}{\sigma_{j1}^{2}\sum_{j}\frac{1}{\sigma_{j1}^{2}}}$$ (12)

where *W*_*j*_ is the weighting value assigned to the *j*^*th*^ SU for data fusion combination. The lower weights are assigned by the FC to the reports of SUs with higher variance under presence hypothesis before the combination. As MUs including always Yes, always No, opposite and random opposite MUs have dissimilar *K*_*j*1_(*i*) and *K*_*j*0_(*i*) in comparison with normal SUs, therefore their contribution in effecting CSS rule is minimized.

### Updating mean and variance for the next iteration

A perfect values of (*μ*_*j*1_, *μ*_*j*0_) and $\left(\sigma_{j0}^{2},\sigma_{j1}^{2}\right)$ for calculating KL divergence is not possible due to unavailability of exact information about the PU. Therefore, universal decision *G*_*B*_(*i*) value calculated previously is further taken as an estimate of the PU signal for calculating and updating mean and variance values, which is used in the next sensing interval for KL divergence value calculation.

$$Z_{j1}=\left\{Z_{j}(i)|H_{1}\right\}\approx \left\{Z_{j}(i)|G_{B}(i)=H_{1}\right\}$$ (13)

$$Z_{j0}=\left\{Z_{j}(i)|H_{0}\right\}\approx \left\{Z_{j}(i)|G_{B}(i)=H_{0}\right\}$$ (14)

Therefore, based on the universal decision results generated by the FC updated values of mean and variances are calculated. If the global decision *G*_*B*_(*i*) = 1, mean and variance *μ*_*j*1_ and $\sigma_{j1}^{2}$ are updated as:

$$\begin{array}{l} \mu_{j1}=D_{1}\mu_{j1}+D_{2}Z_{j}(i)\\ \sigma_{j1}^{2}=D_{1}\sigma_{j1}^{2}+\frac{D_{1}}{D_{2}}{\left[Z_{j}(i)-\mu_{j1}\right]}^{2} \end{array}$$ (15)

Similarly, if *G*_*B*_(*i*) = 0, then mean and variance *μ*_*j*0_, $\sigma_{j0}^{2}$ are updated for all SUs as:

$$\begin{array}{l} \mu_{j0}=D_{1}\mu_{j0}+D_{2}Z_{j}(i)\\ \sigma_{j0}^{2}=D_{1}\sigma_{j0}^{2}+\frac{D_{1}}{D_{2}}{\left[Z_{j}(i)-\mu_{j0}\right]}^{2} \end{array}$$ (16)

$D_{1}=\frac{d}{d-1}$ and $D_{2}=\frac{1}{d}$, where *d* is window size related to the history of the sensing performance for estimated mean and variance.

**Fig 4:**
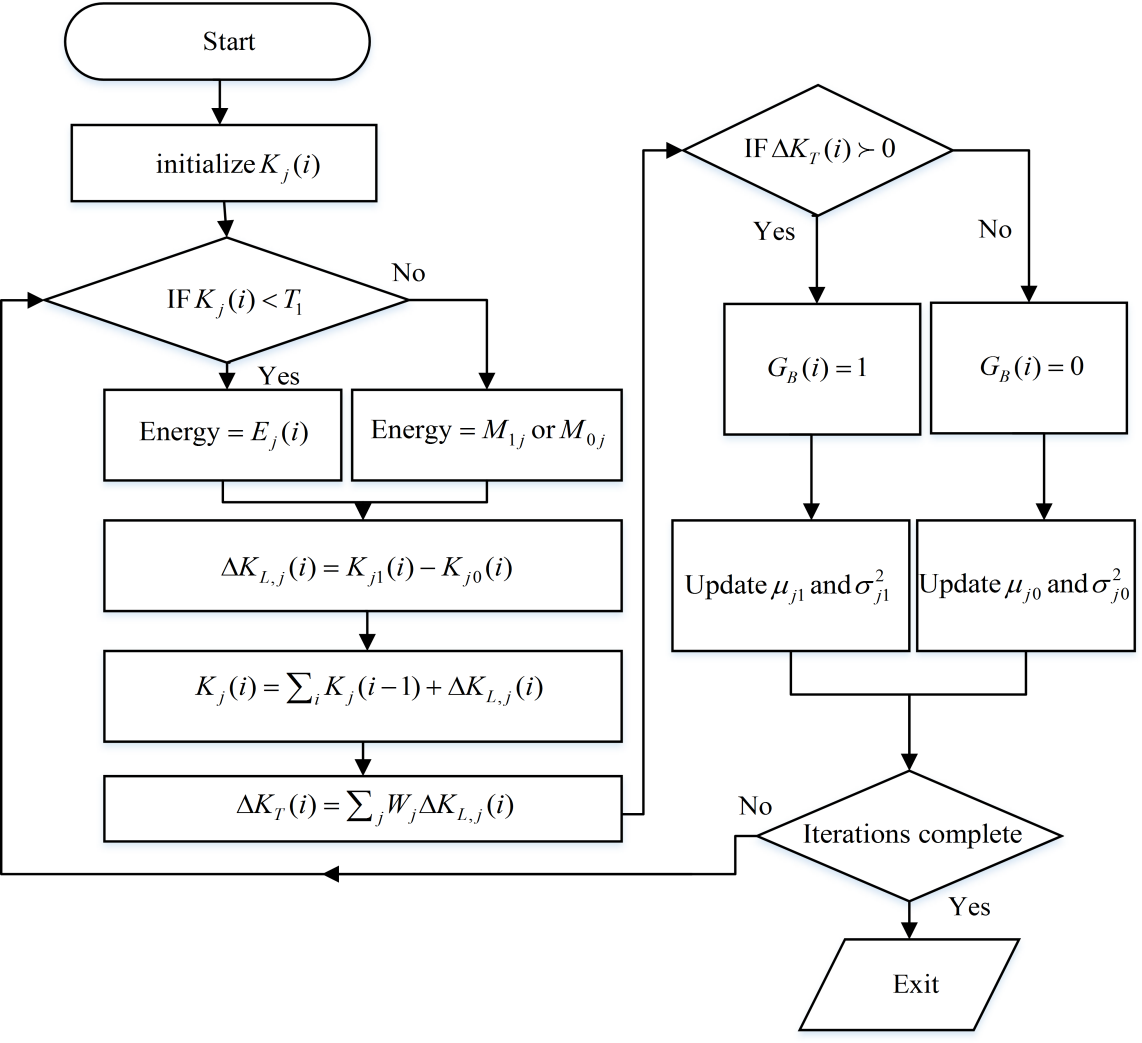
Flowchart diagram of the proposed CSS mechanism.

A flowchart diagram representing the detail operation of the proposed scheme is shown in Fig 4.

## Simulation results and discussions

**Fig 5:**
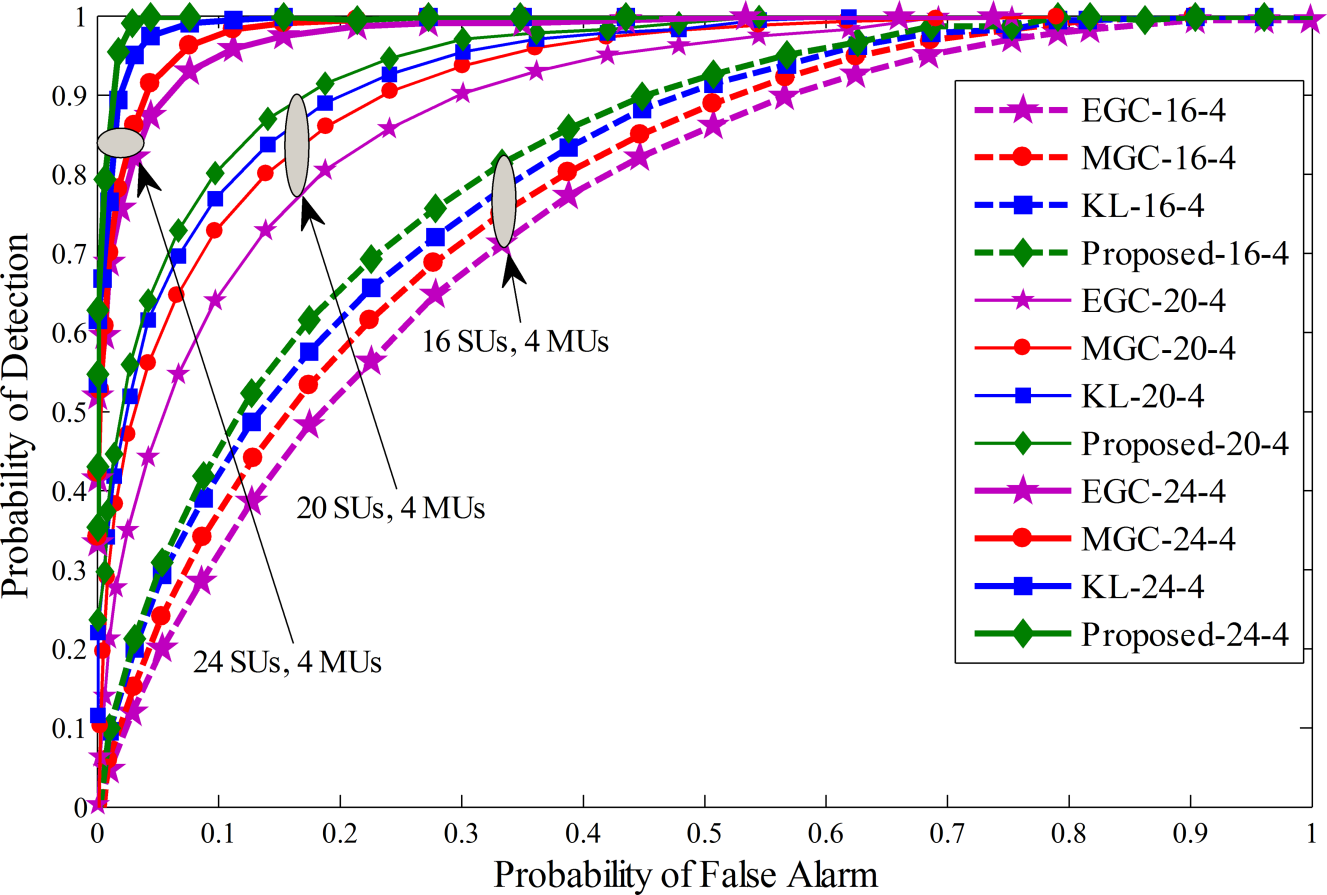
Probability of detection vs probability of false alarm (ROC) curve for (1) 16 total SUs with 4 MUs (2) 20 total SUs with 4 MUs (3) 24 total SUs with 4 MUs.

**Fig 6:**
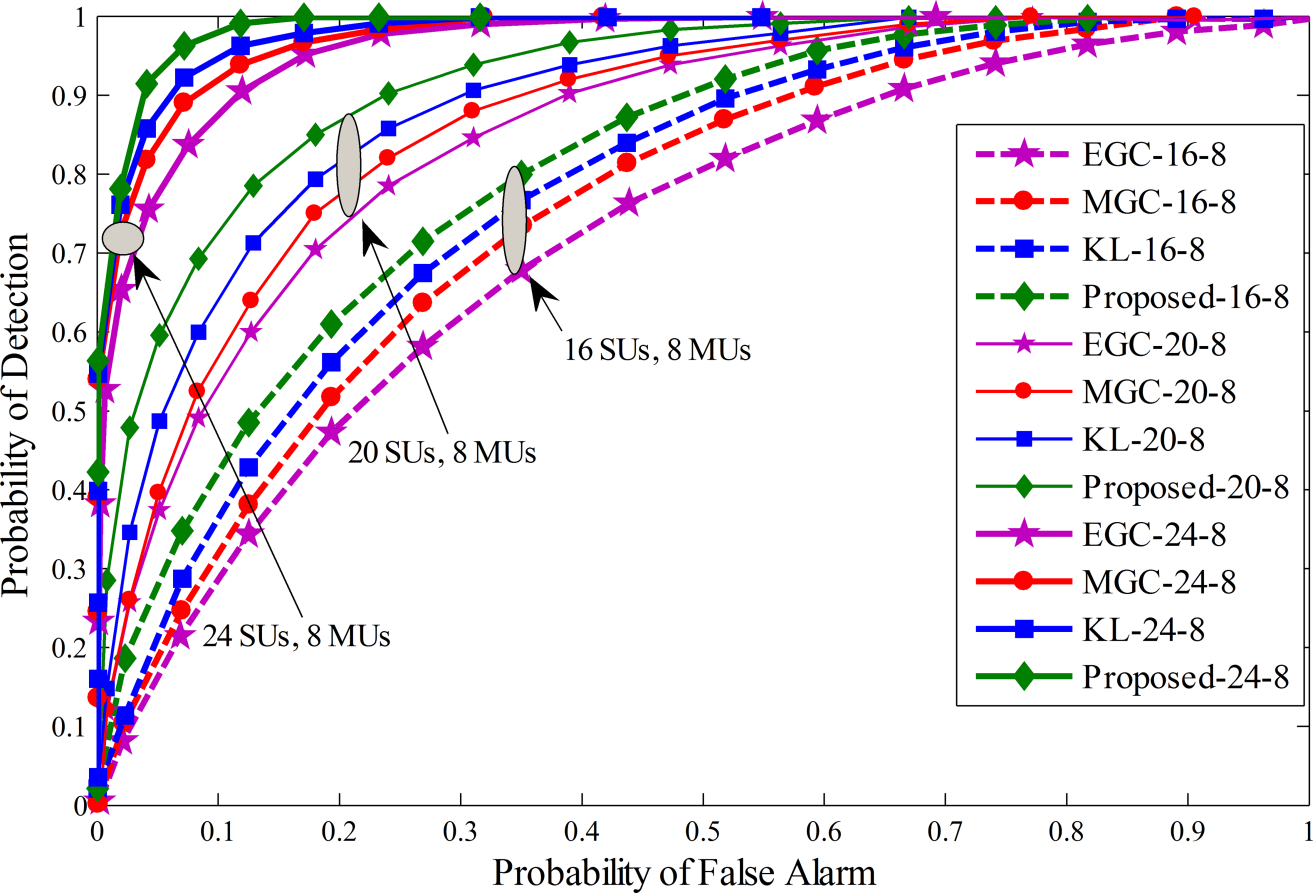
Probability of detection vs probability of false alarm (ROC) for (1) 16 total SUs with 8 MUs (2) 20 total SUs with 8 MUs (3) 24 total SUs with 8 MUs.

**Fig 7:**
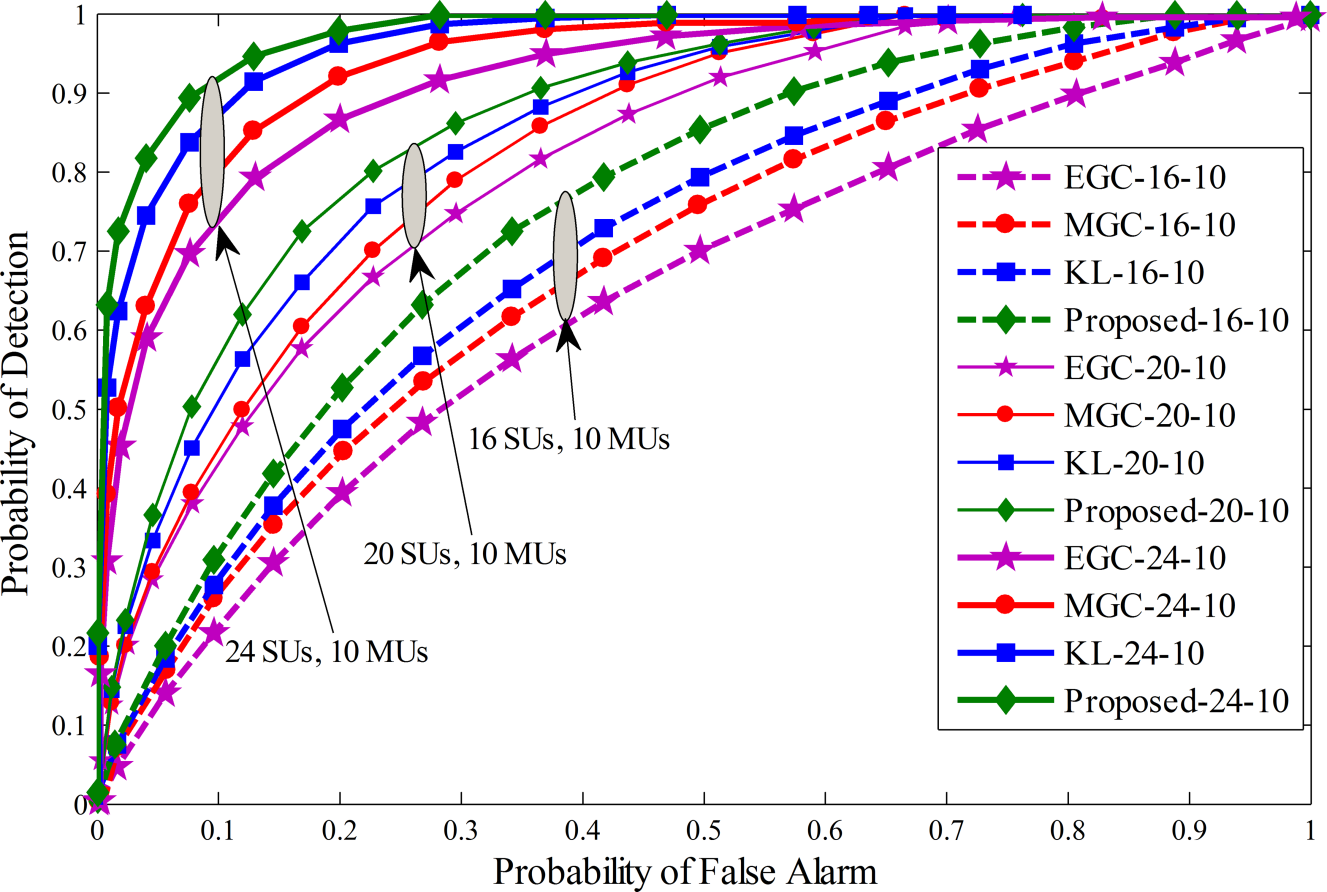
Probability of detection vs probability of false alarm (ROC) for (1) 16 total SUs with 10 MUs (2) 20 total SUs with 10 MUs (3) 24 total SUs with 10 MUs.

**Fig 8:**
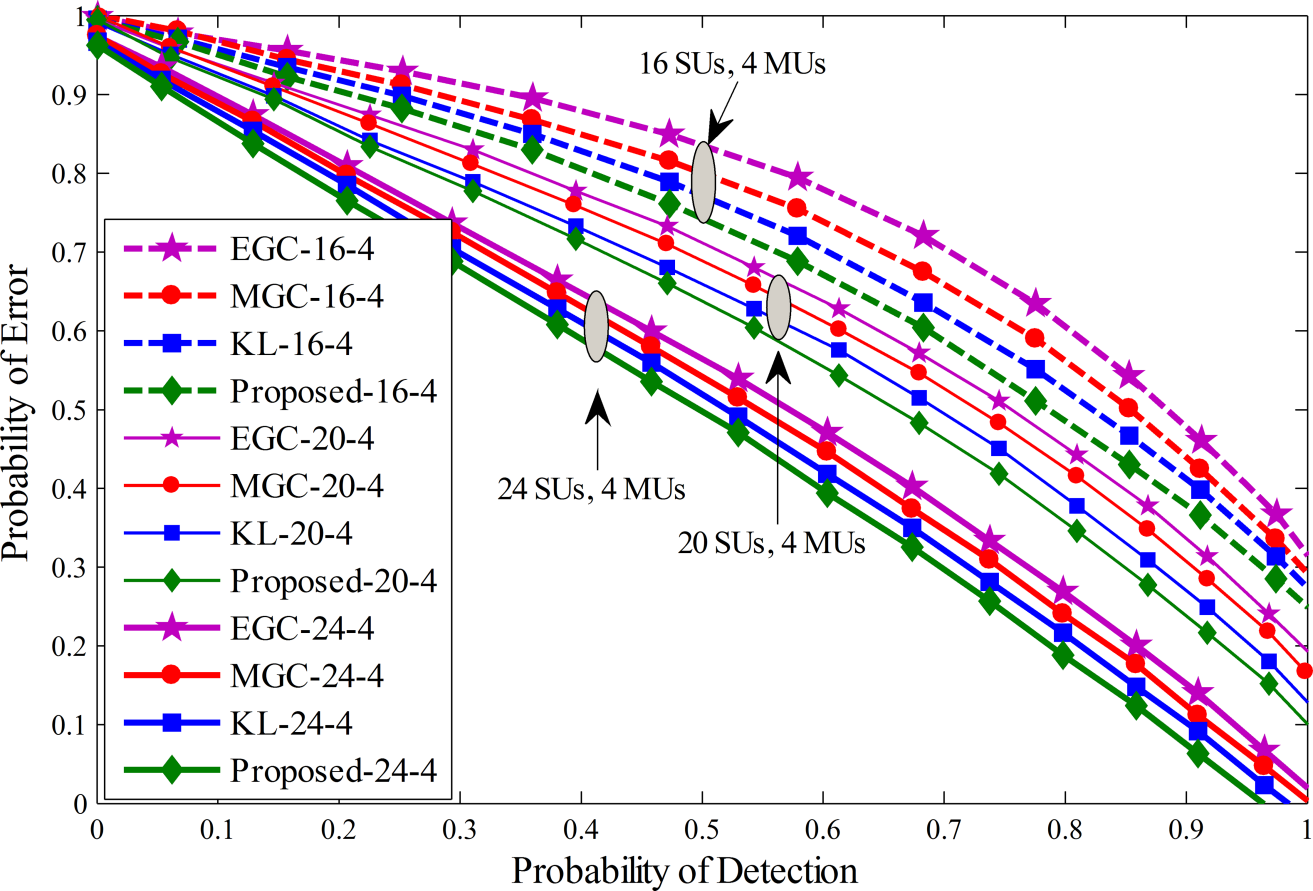
Probability of error vs probability of detection for (1) 16 total SUs with 4 MUs (2) 20 total SUs with 4 MUs (3) 24 total SUs with 4 MUs.

**Fig 9:**
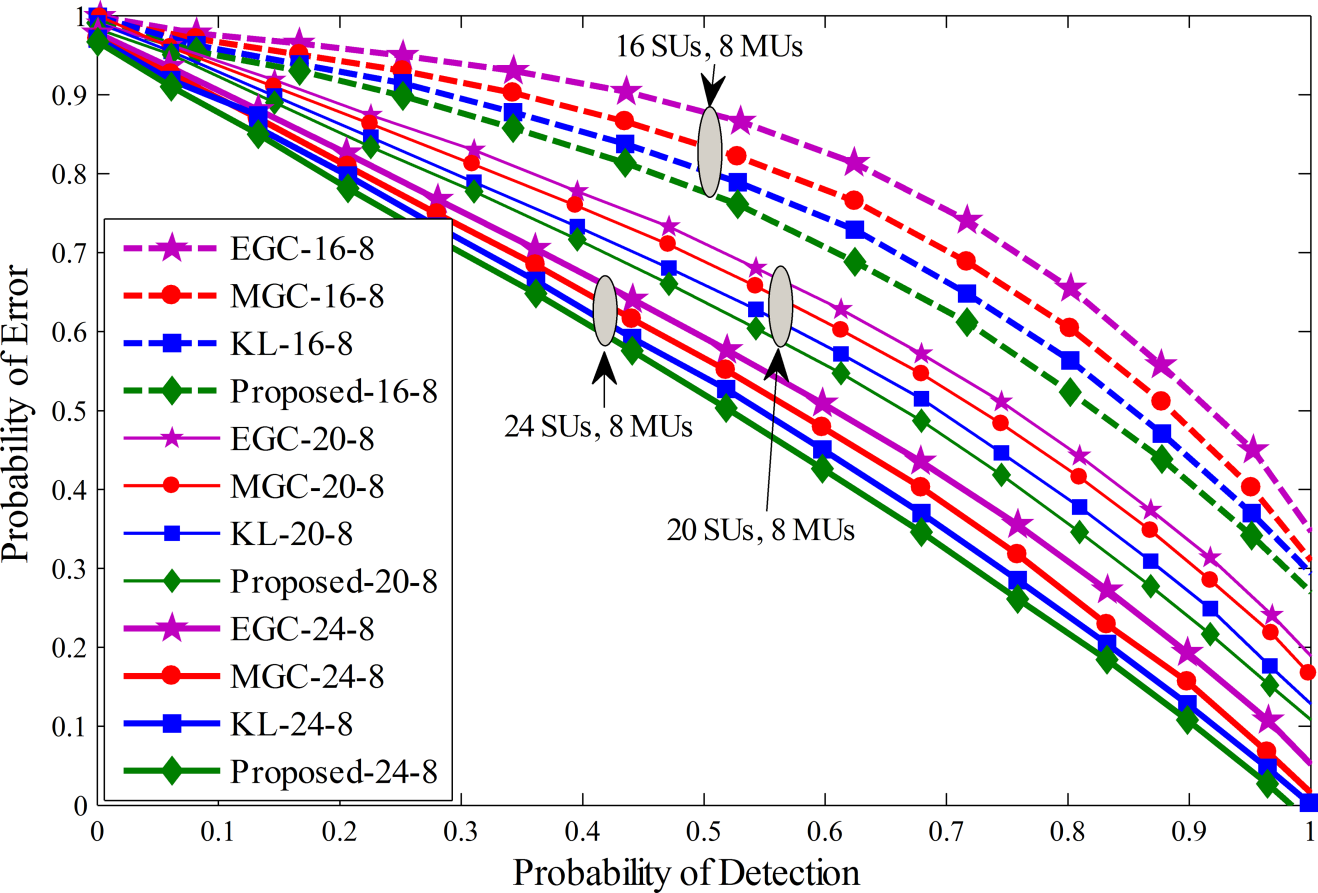
Probability of error vs probability of detection for (1) 16 total SUs with 8 MUs (2) 20 total SUs with 8 MUs (3) 24 total SUs with 8 MUs.

**Fig 10:**
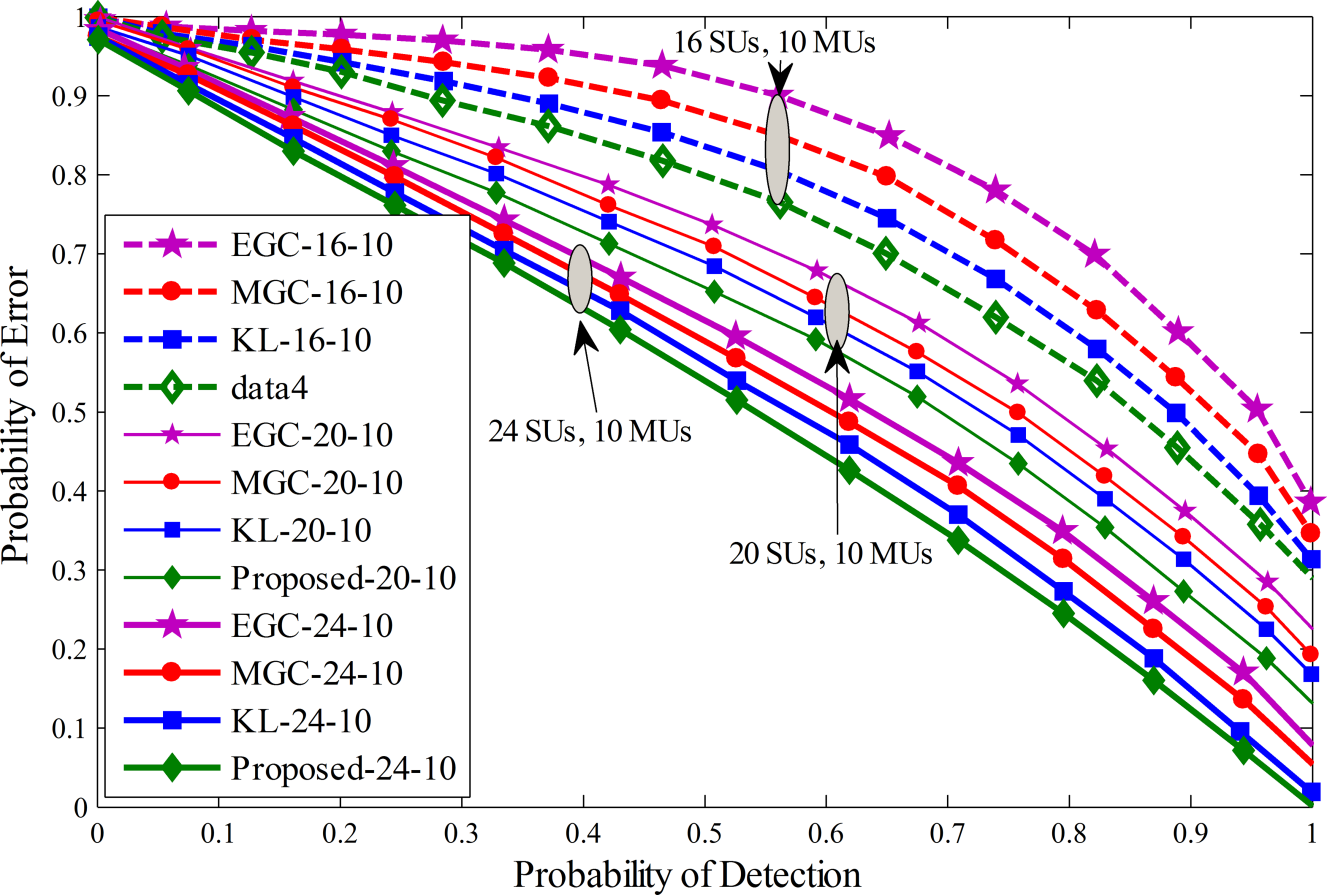
Probability of error vs probability of detection for (1) 16 total SUs with 10 MUs (2) 20 total SUs with 10 MUs (3) 24 total SUs with 10 MUs.

For simulation purposes parameters setting is made for the Cognitive Radio Network with a total number of 16, 20 and 24 SUs at different ratios of MUs. Variation in the SNR for the SUs is made in range of -20 dB to -10 dB. The window size *d* related to the history of the sensing information is kept as 250 and *k* (energy affecting mean variance values) is elected as 25. The sensing time is taken as 1ms for each SU and the number of samples in a sensing interval is taken as *M* = 270. Total sensing iterations *N* in this simulation is 100, and the maximum number of MUs considered for comparison are4, 8 and 10 with equal distributions of always Yes, always No, opposite and random opposite MUs for comparing the detection performance. The system was simulated under three different cases. In the first phase4 MUs are selected in 16, 20 and 24 cooperative SUs with equal percentage of always Yes, always No, opposite and random opposite MUs. Similarly, in the second phase, 8 MUs were equally distributed as always Yes, always No, opposite and random opposite MUs under total 16, 20 and 24 cooperative SUs. In the third phase the number of MUs are extended to 10 for a total of 16, 20 and 24 SUs to test the performance. The proposed scheme is compared with previous KL divergence, maximum gain combination (MGC) and equal gain combination (EGC). ROC curve is drawn for proposed method, traditional KL[12], MGC and EGC schemes in Figs 5–7. Simulation results confirmed that the proposed scheme has better detection results of the PU than the previous KL, EGC and MGC schemes at different levels of total cooperative and MUs. The ROC results collected in these figure show the superiority of the proposed method in comparison with traditional KL, EGC and MGC schemes. Fig 5 result plotted between false alarm and detection probability for a total of 4 MUs when the total number of SUs varies from 16 to 24. It is clear to see that as the total number of SUs increases in Fig 5, the detection results of all fusion schemes increases with increasing total number of cooperative SUs for a given false alarm and fixed number of MUs. Similarly, ROC results are generated for the proposed and all other fusion schemes i.e. traditional KL, EGC and MGC in Figs 6 and 7 with total 8 and 10 MUs at different levels of cooperative SUs. By comparing the results collected in Figs 5–7 it is noticeable to change predominant increase in the probability of detection for a given false alarm probability as the total number of cooperative SUs increases from 16 to 24. The results generated in Figs 5–7 also shows that as the number of total MUs were increased from 4 in Fig 5 to 10 in Fig 7, the proposed method was comparatively less affected with the increasing number of MUs in comparison with other soft fusion schemes. In this part of the simulation results the proposed method is able to provide higher detection results for a given false alarm at different concentration levels of cooperative and MUs including always Yes, always No, opposite and random opposite category of MUs.

A similar comparison is shown in Figs 8–10 by drawing the probability of error against probability of detection for the proposed, KL [12], MGC and EGC schemes. The graphical results showed improved detection results for the proposed scheme against traditional KL, MGC and EGC schemes at all number of cooperative and malicious SUs. By the inspection of these results, it is noticeable that the error in terms of detection of the licensed user for the proposed scheme decreases more quickly as compared with previous fusion schemes and has less vulnerability to the introduction of increasing MUs.

**Fig 11:**
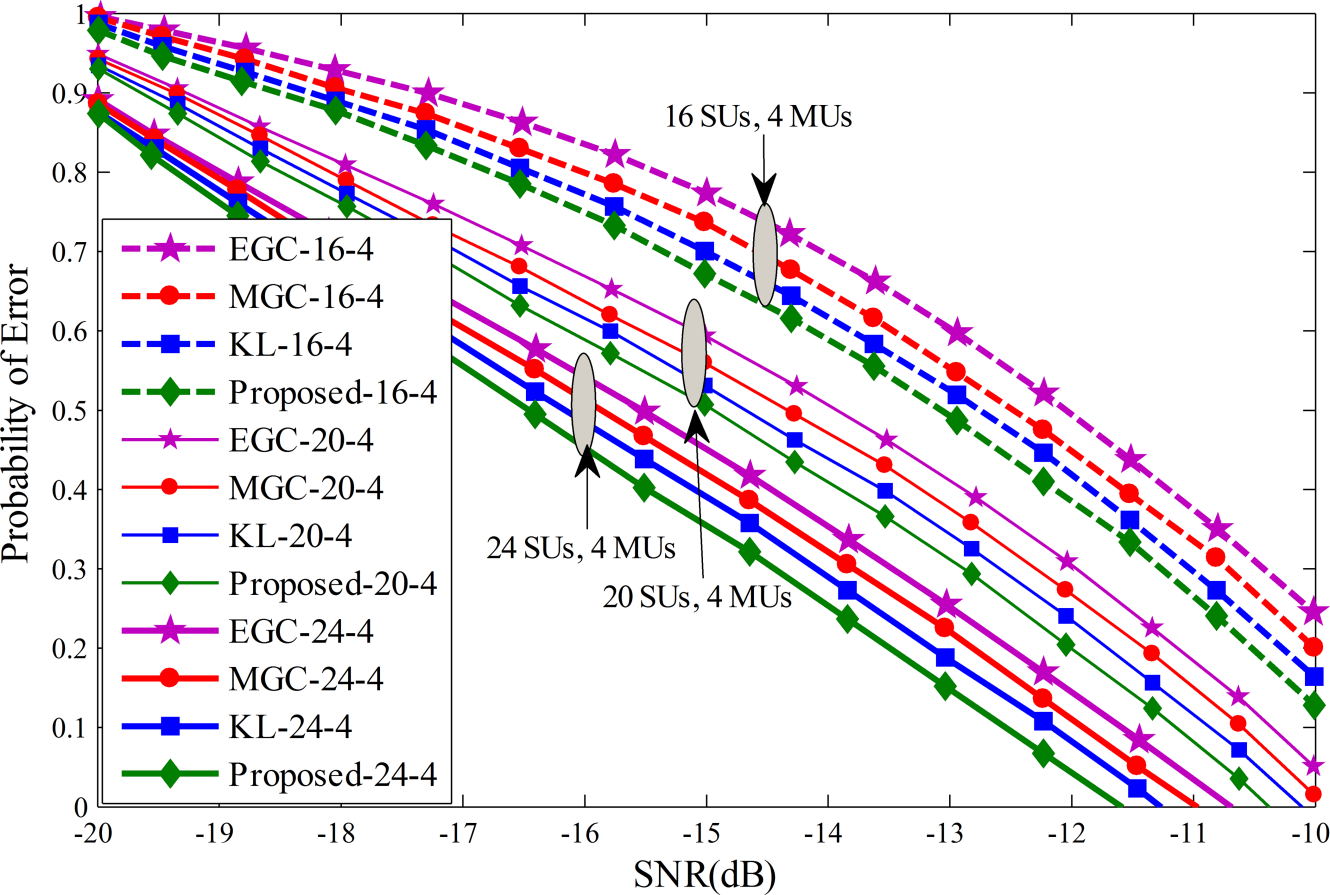
Probability of error vs signal to noise ratio for (1) 16 total SUs with 4 MUs (2) 20 total SUs with 4 MUs (3) 24 total SUs with 4 MUs.

**Fig 12:**
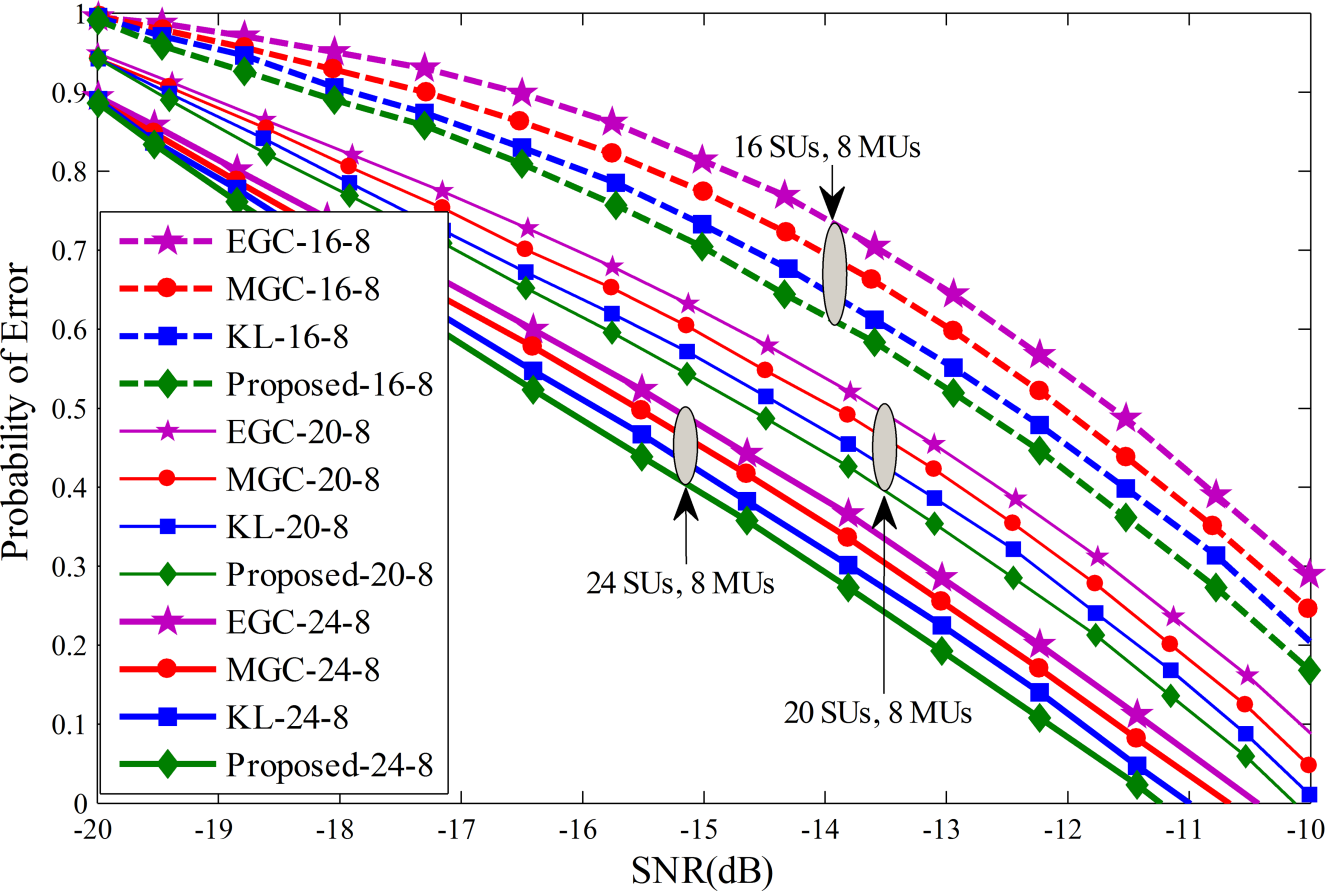
Probability of error vs signal to noise ratio for (1) 16 total SUs with 8 MUs (2) 20 total SUs with 8 MUs (3) 24 total SUs with 8 MUs.

**Fig 13:**
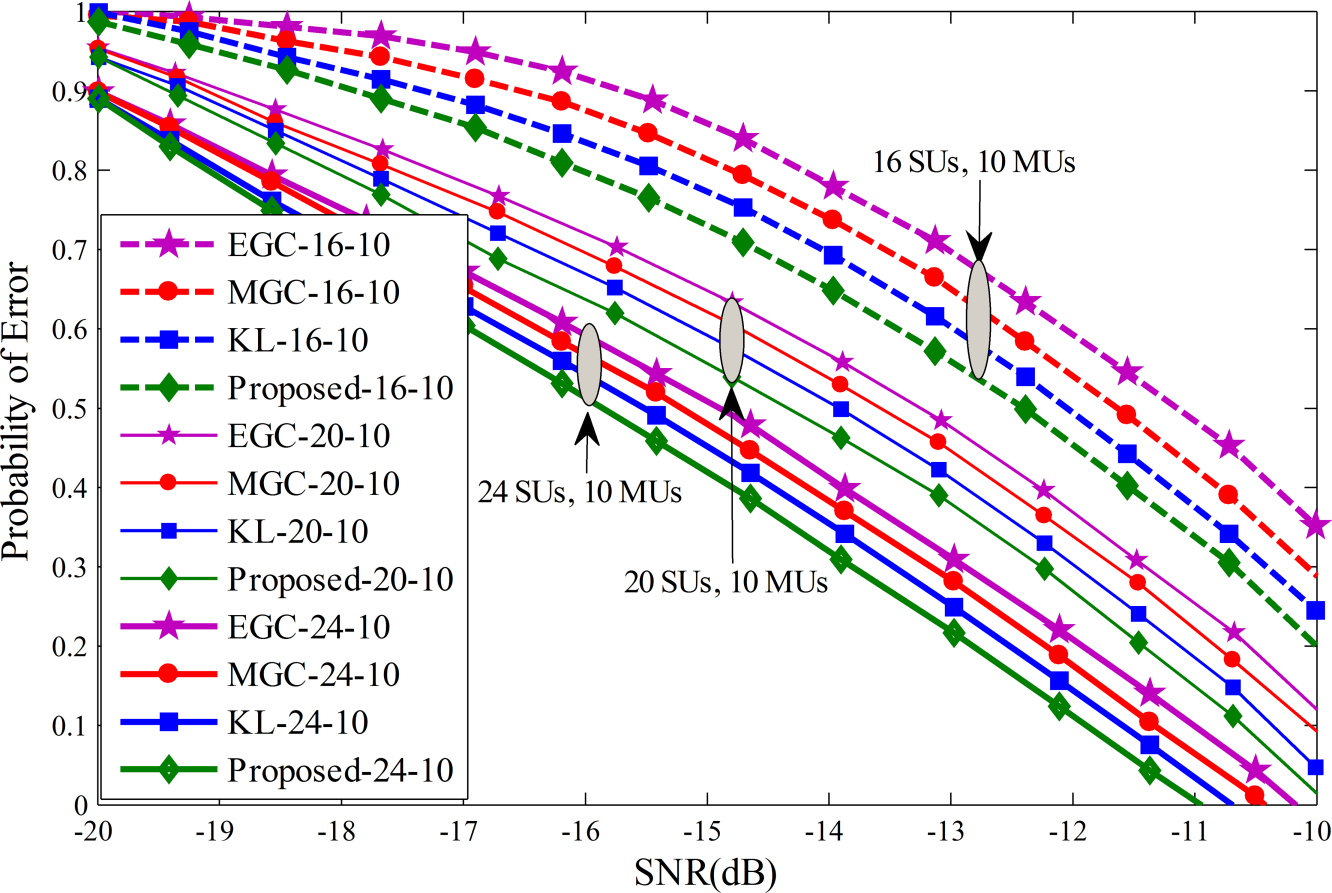
Probability of error vs signal to noise ratio for (1) 16 total SUs with 10 MUs (2) 20 total SUs with 10 MUs (3) 24 total SUs with 10 MUs.

Probability of error results for each individual SU is drawn against the SNRs varying from -20 dB to -10 dB in Figs 11–13. The graphical results showed that with the increasing average SNR values, the proposed method results showed sophisticated improvements and is able to reduce the channel sensing error quickly in comparison with all other fusion schemes. Similarly, it can be seen that for a given average SNR value, the probability of error decrease even further by varying the total number of cooperative SUs from 4 to 10 in Figs 11–13. The efficiency in terms of sensing the licensed user channel reduces with an increase in the total number of MUs from 4 in Fig 11 to 10 in Fig 13 at different level of total SUs. The proposed method results in these plots is less effected with the increase number of MUs and demonstrate healthier results of the proposed scheme among all, while EGC is having worst probability of error results.

**Fig 14:**
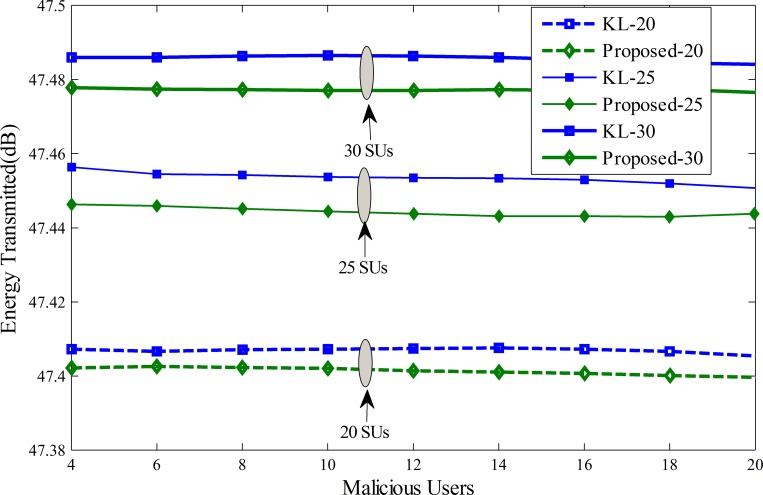
Energy trasmitted vs number of MUs for (1) total 20 SUs (2) total 25 SUs (3) total 30 SUs.

For energy comparison of the proposed scheme and KL[12], simulation results are plotted in Fig 14 among the total average transmitted energy of all SUs and MUs. In Fig 14 the total number of MUs is increased from 4 to 20 and the average transmitted energy of all SUs are collected under 20, 25 and 30 total cooperative SUs. It is obvious from Fig 14 that the proposed scheme is outperforming the traditional KL method in terms of energy utilization in all three cases when 20, 25 and 30 cooperative SUs participate in CSS. The energy transmitted by all SUs increases when the number of cooperative SUs is increased from 20 to 30for a given total number of MUs. The MUs are selected for energy comparison with 25% always Yes, 25% always No, 25% opposite and 25% random opposite MUs. These energy plots display that the proposed scheme results in overall savings of the transmitting energy for the proposed scheme under all 20, 25 and 30 total cooperative SUs. The simulation results show effectiveness of the proposed scheme in getting higher detection results of the PU, which results in lower error with optimize transmission energy.

## Conclusion

As MUs misinform other SUs about the PU spectrum, it is therefore mandatory to withdraw information provided by MUs in the CSS environment. KL divergence is a tool used for the detection of MU based on the PDF dissimilarity of a normal user and MUs in CSS. The proposed scheme is using the KL divergence with a modified pre-sensing check for each SU before forwarding the observed spectrum information to the FC. The SUs with reputation score in the form of KL divergence feedback by the FC attained, will report FC about the PU status with sensed energy based on the current and past results from its local database. Simulation results demonstrate the effectiveness of the proposed scheme in terms of sophisticated detection while exercising comparatively less total transmission energy.

This study has limited analysis of the different fusion schemes in the presence of always Yes, always No, opposite and random opposite MUs for sensing merely one PU spectrum. The proposed scheme could be further enhanced for sensing more than one PU spectrum by introducing primary user emulation attack category of MU, resembling the behavior of the PU to misguide other SUs. Similarly, the behavior of the proposed technique can be checked with other schemes by assigning lower and higher SNR values to MUs in comparison with normal SUs to confirm if this method is able to identify and separate MUs under low and higher SNRs.

The KL divergence scheme adopted in this work for identifying MUs is based on the energy distribution of the individual SU report. In future, the KL divergence measurement will be estimated based on sensed information of an individual SU with the average of the sensing information provided by all other SUs. A reputation of the KL divergence for individual SUs can be used in the subsequent sensing intervals to avoid sensing information from the confirmed MUs.
